# Cerium Oxide Nanoparticles Sensitize Pancreatic Cancer to Radiation Therapy through Oxidative Activation of the JNK Apoptotic Pathway

**DOI:** 10.3390/cancers10090303

**Published:** 2018-09-01

**Authors:** Melissa S. Wason, Heng Lu, Lin Yu, Satadru K. Lahiri, Debarati Mukherjee, Chao Shen, Soumen Das, Sudipta Seal, Jihe Zhao

**Affiliations:** 1 Burnett School of Biomedical Sciences University of Central Florida College of Medicine, Orlando, FL 32827, USA; Melissa.Wason@ucf.edu (M.S.W.); Heng.Lu@ucf.edu (H.L.); Lin.Yu@ucf.edu (L.Y.); Satadru.Lahiri@ucf.edu (S.K.L.); Debarati.Mukherjee@ucf.edu (D.M.); Chao.Shen@ucf.edu (C.S.); 2 Department of Surgery, Miller School of Medicine, University of Miami, Miami, FL 33136, USA; 3 Cardiovascular Research Institute, Department of Molecular Physiology & Biophysics, Baylor College of Medicine, Houston, TX 77030, USA; 4 Department of Pharmacology and Cancer Biology, Duke University School of Medicine, Durham, NC 27710, USA; 5 Department of Microbiology, College of Life Sciences, Wuhan University, Wuhan, Hubei 430072, China; 6 Department of Mechanical, Materials and Aerospace Engineering, Advanced Materials Processing and Analysis Center; Nanoscience and Nanotechnology Center, University of Central Florida, Orlando, FL 32816, USA; Soumen.Das@ucf.edu (S.D.), Sudipta.Seal@ucf.edu (S.S.)

**Keywords:** cerium oxide nanoparticles, radiation therapy, cancer, thioredoxin 1, JNK, apoptosis

## Abstract

Side effects of radiation therapy (RT) remain the most challenging issue for pancreatic cancer treatment. Cerium oxide nanoparticles (CONPs) are currently being tested in pre-clinical trials as an adjuvant to sensitize pancreatic cancer cells to RT and protect normal tissues from the harmful side effects. CONPs were not able to significantly affect RT-induced DNA damage in cancer cells, thereby ruling out sensitization through increased mitotic catastrophe. However, activation of c-Jun terminal kinase (JNK), a key driver of RT-induced apoptosis, was significantly enhanced by co-treatment with CONPs and RT in pancreatic cancer cells in vitro and human pancreatic tumors in nude mice in vivo compared to CONPs or RT treatment alone. Further, CONP-driven increase in RT-induced JNK activity was associated with a marked increase in Caspase 3/7 activation, indicative of apoptosis. We have previously shown that CONPs increase reactive oxygen species (ROS) production in cancer cells. ROS has been shown to drive the oxidation of thioredoxin 1 (TRX1) which results in the activation of apoptosis signaling kinase 1 (ASK1). The increase in ASK1 activation following the co-treatment with CONPs followed by RT suggests that the increased JNK activation is the result of increased TRX1 oxidation. The ability of CONPs to sensitize pancreatic cancer cells to RT was mitigated when the TRX1 oxidation was prevented by mutagenesis of a cysteine residue or when the JNK activation was blocked by an inhibitor. Taken together, these data demonstrate an important mechanism for CONPs in specifically killing cancer cells and provide novel insights into the utilization of CONPs as a radiosensitizer and therapeutic agent for pancreatic cancer.

## 1. Introduction

One in two men and one in three women in the United States will develop cancer at some point in their lifetimes [1]. Of the nearly 600,000 cancer related deaths in the USA each year [2], roughly 30,000 of those deaths are caused by pancreatic cancer [3]. Only 15–20% of pancreatic cancer patients present with surgically resectable disease and roughly 20% of patients undergoing surgical resection survive five years post-operation [3]. The main treatment suggested for patients with locally advanced, or surgically unresectable, pancreatic cancer is a combination of chemotherapy and radiation therapy [3]. Chemoradiation has been shown to both extend survival and decrease the pain commonly associated with pancreatic cancer [3]. However, current chemotherapeutic and radiotherapeutic agents for advanced pancreatic cancer have shown minimal lasting impact, as most patients show signs of progression and metastatic development within only a few months of completing treatment [3].

It is known that radiation therapy (RT) kills cells via multiple mechanisms, mainly mitotic catastrophe and apoptosis [4]. In cell types that are prone to apoptotic cell death, such as hematopoietic cells, apoptosis is the predominant cell death pathway induced by exposure to radiation [4]. However, most solid human malignancies, including pancreatic cancer, generally lose pro-apoptotic mechanisms over the course of tumor progression [4]. As a result, mitotic catastrophe becomes the alternative, driving mechanism of cell death induced by RT in solid tumors, especially of epithelial origin [5].

Mitotic catastrophe, or clonogenic cell death, occurs when unrepaired DNA damage does not properly arrest the cell cycle [6]. Just as many malignant cells lose pro-apoptotic mechanisms, they often also become deficient in cell cycle checkpoints during transformation and progression, which contributes to the chance for mitotic catastrophe [6]. The premature progression of the cell cycle through mitosis results in the abnormal division of chromosomes during cell division, often leading to giant cells with unusual nuclear morphology, multiple nuclei, or multiple micronuclei [4]. The nuclear and chromosomal abnormalities eventually kill the cell through delayed apoptosis or necrosis.

Apoptosis, or programmed cell death, is an essential cellular process for the development and maintenance of normal cells and tissues in the body [7]. Malfunctions in the apoptotic machinery have been linked to a variety of diseases, including cancer. As cells move through the neoplastic progression, they lose sensitivity to many of the apoptotic stimuli, which may help explain why cancers in the early stages tend to be more sensitive to chemotherapy and radiotherapy [7]. Therefore, especially for cancers such as pancreatic cancer that are commonly diagnosed in late stages, it is critical to identify treatments that are able to overcome the resistance of cancer cells to the available treatment modalities.

Cerium oxide nanoparticles (CONPs) are a new compound currently being pursued in pre-clinical trials for their potential use in the treatment of cancer. Originally tested for their ability to scavenge radicals and protect normal tissues from radiation-induced damage associated with treating cancer in the head and neck [8], intestine [9], lung [10], and breast [11], the application of CONPs has expanded beyond the mitigation of the side effects of other cancer therapies. Several reports have documented the inherent toxicity of CONPs to cancer cells of various origins, including alveolar epithelial cancer cells [12], hepatocellular carcinoma cells [13], and pancreatic carcinoma cells [14]. One study described that CONPs can directly inhibit squamous carcinoma cell invasion, as well as indirectly inhibit invasion by blocking the formation of myofibroblasts [15]. Other researchers have demonstrated CONP-induced inhibition of the growth and invasion in melanoma cells [16]. A recent publication also established the anti-angiogenic properties of CONPs in an ovarian cancer model [17]. The testing of CONP-based cancer therapies is rapidly expanding, both as the primary treatment and as an adjuvant treatment for already established therapies [18].

Our previous publication demonstrates that CONPs possess inherent toxicity and the ability to sensitize pancreatic cancer cells to radiation-induced cell death, without negatively impacting the corresponding normal cell viability or radiosensitivity [14]. CONPs were also shown to selectively increase RT-induced ROS production in pancreatic cancer cells [14].

Herein, we identified one of the redox-responsive pathways to be responsible for the radiosensitization of pancreatic cancer cells by CONPs. Through activating this pathway, CONPs rescue the apoptotic mechanism and RT-sensitivity in the otherwise RT-resistant pancreatic cancer cells.

## 2. Results

### 2.1. CONPs Do Not Impact RT-Induced DNA Damage in Pancreatic Cancer Cells but Inhibit It in Normal Pancreatic Epithelial Cells

**Figure 1:**
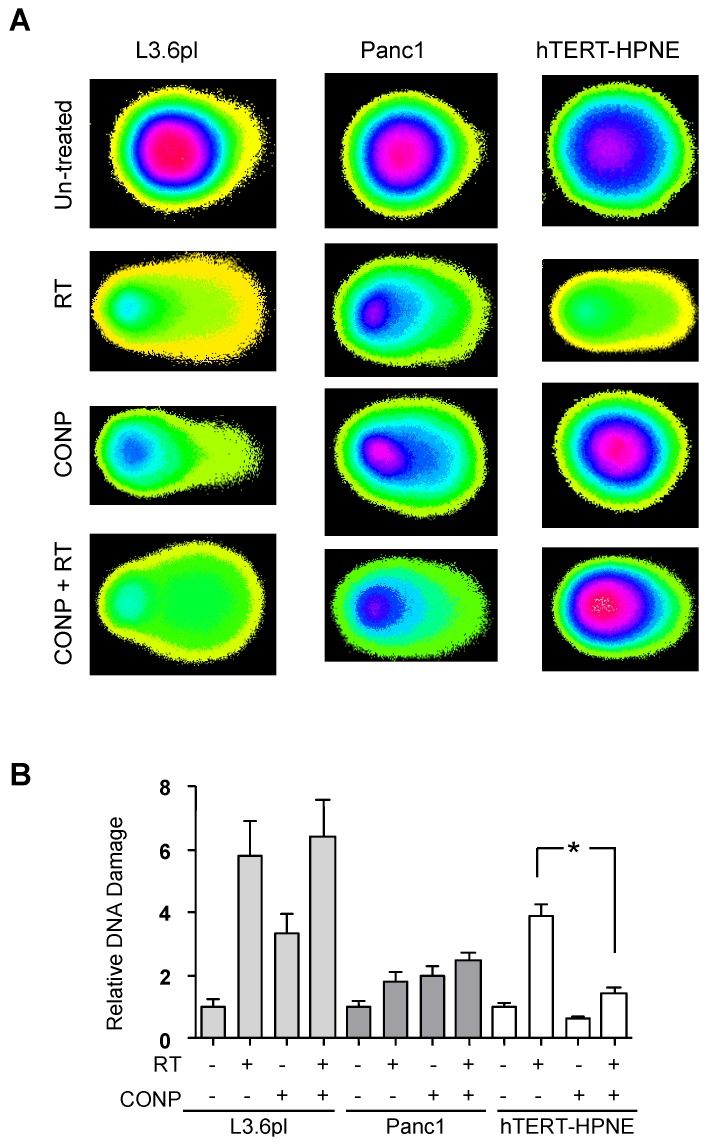
CONPs Do Not Sensitize Pancreatic Cancer Cells to RT via DNA Damage. Cells were treated with 10 μM CONPs followed by exposure to 0 or 5 Gy RT 24 h later. Immediately after RT, cells were collected for Comet assay. (**A**) L3.6pl and Panc1 cells show DNA damage resulting from RT, but CONPs did not significantly alter DNA damage patterns, whereas hTERT-HPNE cells were protected from RT-induced DNA damage by pre-treatment with CONPs. (**B**) At least 50 cells per condition were analyzed and graphed relative to the respective (untreated) control. * *p* < 0.001.

To determine if CONPs sensitize pancreatic cells to RT by enhancing mitotic catastrophe, DNA damage analysis was performed. L3.6pl and Panc1 pancreatic cancer cells exposed to RT, CONPs, or the combination of CONPs followed by RT. They all displayed some degree of DNA damage, as indicated by the ratio of the amount of DNA in the comet head to the amount of DNA in the comet tail (Figure 1A,B).

However, the combination therapy did not display additive effects; namely, CONPs followed by RT did not induce a greater amount of DNA damage than RT alone. Panc1 cells, acknowledged to be more radiation resistant, showed less DNA damage demonstrated by the migration of DNA into the tail, than L3.6pl, which is a comparatively radiation-sensitive cell line. This indicates that the amount of DNA damage that resulted from exposure to RT correlated with the sensitivity of the cancer cell line to radiation-induced death. As this assay was completed immediately following RT exposure, the effects of DNA repair in response to CONP treatment can be excluded. These results suggest that CONP-induced RT-sensitization does not involve the mitotic catastrophe mechanism. In contrast to the lack of effect of CONPs on RT-induced DNA damage in pancreatic cancer cells, normal pancreatic epithelial hTERT-HPNE cells were responsive to pre-treatment with CONPs prior to RT (Figure 1). The cells that were pre-treated with 10 µM CONPs before exposure to 5 Gy RT displayed significantly less DNA damage, indicated by the tail moment, compared to cells exposed to RT alone immediately following RT exposure. Further, CONPs alone did not induce any DNA damage in normal cells. These results indicate that CONPs in fact protect the normal cell from suffering mitotic catastrophe when exposed to RT.

### 2.2. CONPs Drive RT-Induced JNK Activation in Cancer Cells

**Figure 2:**
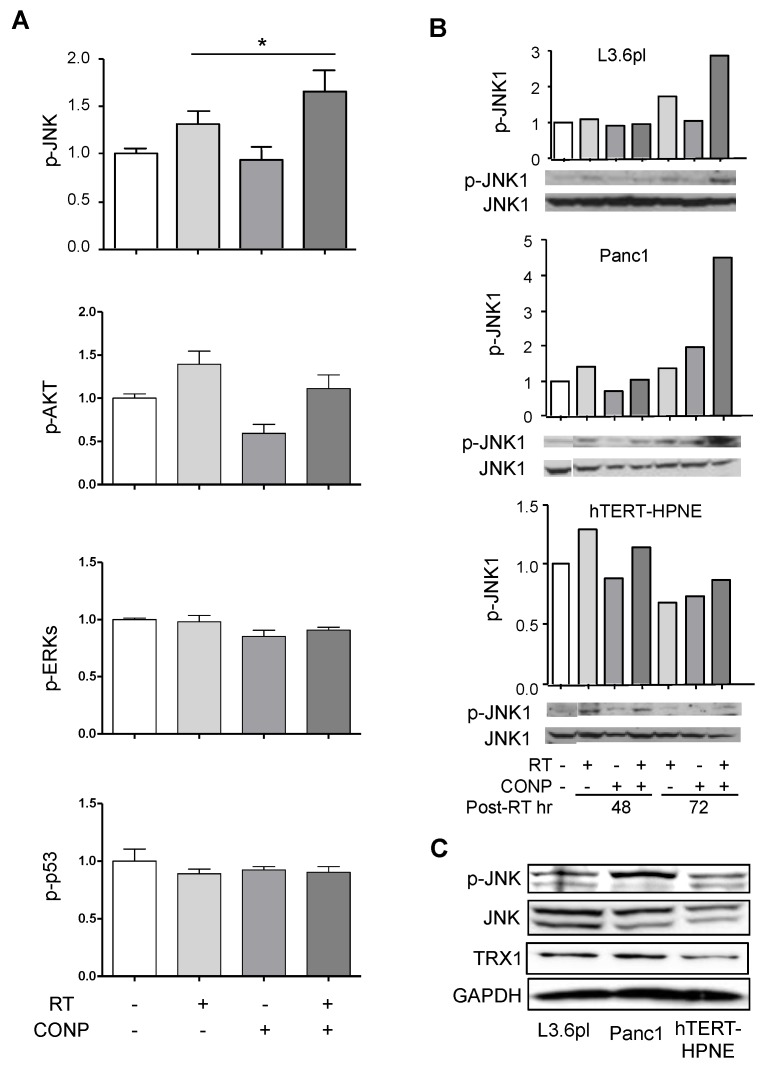
CONP Pre-Treatment Drives RT-Induced JNK Activation in Pancreatic Cancer Cells. (**A**) CONP treatment led to activation of JNK, but not AKT, ERKs or p53 in L3.6pl cells. Cells were treated with 10 μM CONPs for 24 h followed by exposure to 0 or 5 Gy RT and allowed for 72 h prior to PhosphoELISAarray analysis. Levels were normalized to untreated group. * *p* < 0.05. (**B**) Indicated cells were treated similarly for the period of time indicated and subject to p-JNK1 analysis by Western blot (See Figure S1 for similar change in p-JNK2 levels). (**C**) Basal expression of pJNK, JNK, and TRX was determined in the cells by western blot, with cancer cells displaying higher levels of all three proteins.

To determine which pathway is activated in response to RT in cells pre-treated with CONPs, a multi-analyte ELISA for activation of proteins in key pathways associated with human cancer cell survival was performed on L3.6pl human pancreatic cancer cells exposed to CONPs, RT, and the combination therapy. Of the screened targets, only JNK activation was significantly increased by the combination of CONPs followed by RT, none of the other targets (AKT, Erk1/2, p53) displayed any alteration in response to the pre-treatment with CONPs compared to RT alone (Figure 2A).

The ability of CONPs to drive RT-induced JNK activation, as determined by JNK phosphorylation, was further assessed by western blot analysis. The activation of both JNK1 (Figure 2B) and JNK2 (Supplementary Figure S1) was significantly increased in both L3.6pl and Panc1 pancreatic cancer cell lines by 72 h following exposure to RT. In contrast, pre-treatment with CONPs induced a slight decrease or no change in JNK phosphorylation in the hTERT-HPNE normal pancreatic epithelial cells. CONPs alone did not induce significant JNK phosphorylation in either the pancreatic cancer cells or the normal epithelial cells.

When basal expression and phosphorylation of JNK in untreated cells were determined, both L3.6pl and Panc1 pancreatic cancer cell lines had higher total JNK expression than the corresponding normal hTERT-HPNE cells (Figure 2C). Based on the ratio of phosphorylated JNK (p-JNK) to total JNK, nearly all of the JNK present in normal pancreatic epithelial cells was phosphorylated, i.e., activated. However, there was some difference in amount of phosphorylated and total JNK in pancreatic cancer cells, indicating that not all of the JNK present is activated in un-treated pancreatic cancer cells. Interestingly, ratio of active to total JNK correlated with cellular expression of thioredoxin 1 (TRX1), a redox sensing protein upstream of JNK. TRX1 overexpression by cancer cells compared to the corresponding normal cells was confirmed by western blot. Additionally, the normal cells in which a greater percentage of the basal JNK is phosphorylated express lower levels of TRX1 compared to the pancreatic cancer cells which expressed higher levels of TRX1 and lesser degree of basal JNK phosphorylation. These results suggest a possibility that the TRX1-JNK pathway could play a role in CONPs-induced pancreatic cancer cell sensitization to RT.

### 2.3. CONP-Driven JNK Activation Mediates RT-Induced Apoptosis in Pancreatic Cancer Cells

**Figure 3:**
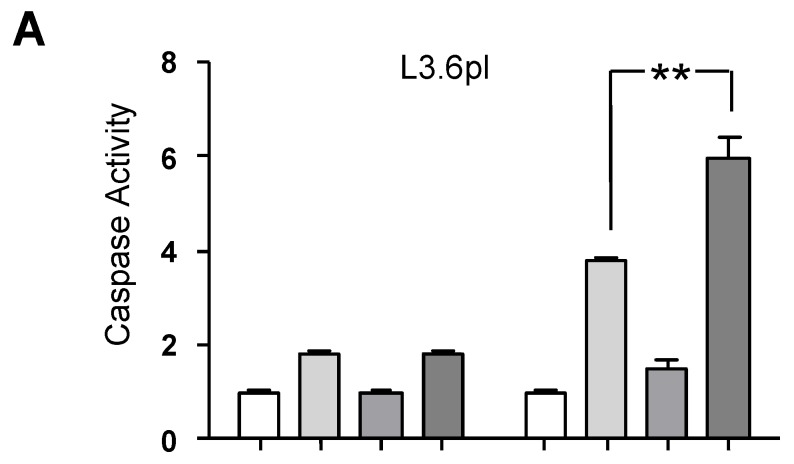

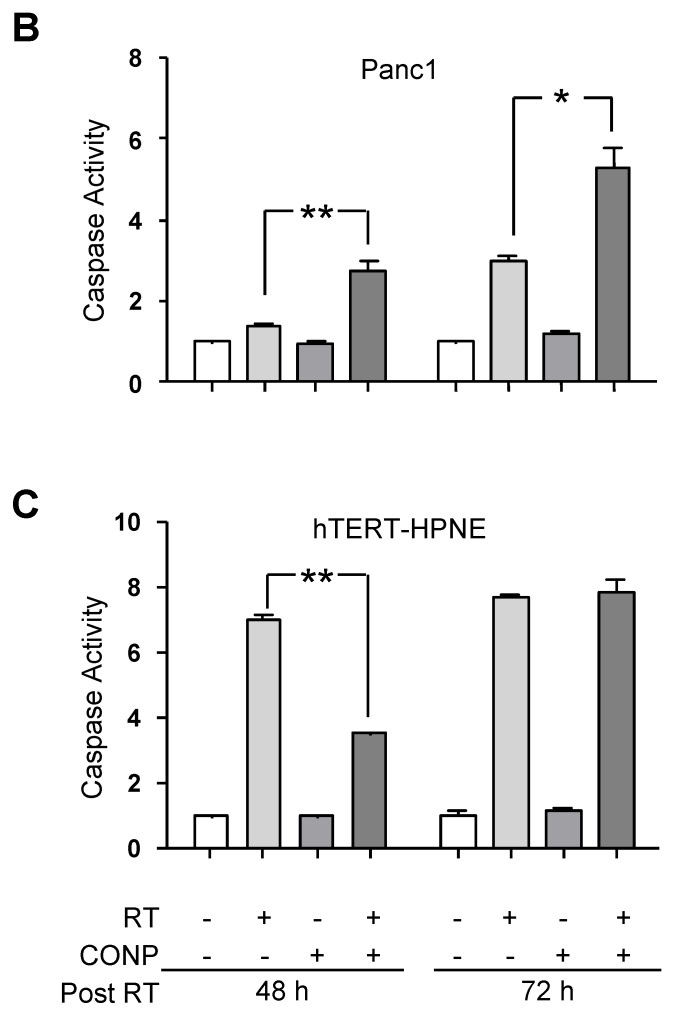
CONP Treatment Drives RT-induced Apoptotic Caspases Activation in Pancreatic Cancer Cells but Not Normal Cells in vitro. L3.6pl (**A**), Panc1(**B**) and hTERT-HPNE (**C**) cells were treated with or without 10 μM CONPs for 24 h followed by exposure to 0 or 5 Gy RT. Caspase 3/7 activation was then determined 48 or 72 h post RT by Caspase Glo assay. Levels were normalized to untreated group. * *p* < 0.05, ** *p* < 0.01.

As JNK activation can be both pro- and anti-apoptotic, we next analyzed caspase 3 and caspase 7 activation as markers of apoptotic cells in vitro. Caspase 3/7 activation increases over time in response to RT in both L3.6pl and Panc1 pancreatic cancer cells. In L3.6pl cells, which are inherently more sensitive to RT-induced cell death, CONP-enhanced caspase activation is not evident until 72 h post RT (Figure 3A). However, in Panc1 cells which are inherently more resistant to RT-induced cell death, the CONP-induced increase apoptotic signaling is discernible by 48 h post RT and persists through 72 h post RT (Figure 3B). The data indicate enhanced, stable activation of apoptosis in both pancreatic cancer cell lines by the combination therapy.

Conversely, CONPs appear to inhibit or delay caspase activation in normal pancreatic hTERT-HPNE cells. At 48 h post RT, caspase 3 and caspase 7 activation are significantly decreased in cells pre-treated with CONPs compared to cells treated with RT alone (Figure 3C). Even at 72 h post RT, there is no increase in the caspase activation in cells treated with CONPs prior to RT compared to RT alone. These results indicate that the CONPs enhance apoptosis in the RT-treated pancreatic cancer cells selectively.

### 2.4. CONPs Drive RT-Induced ASK1 Activation in Pancreatic Cancer Cells

**Figure 4:**
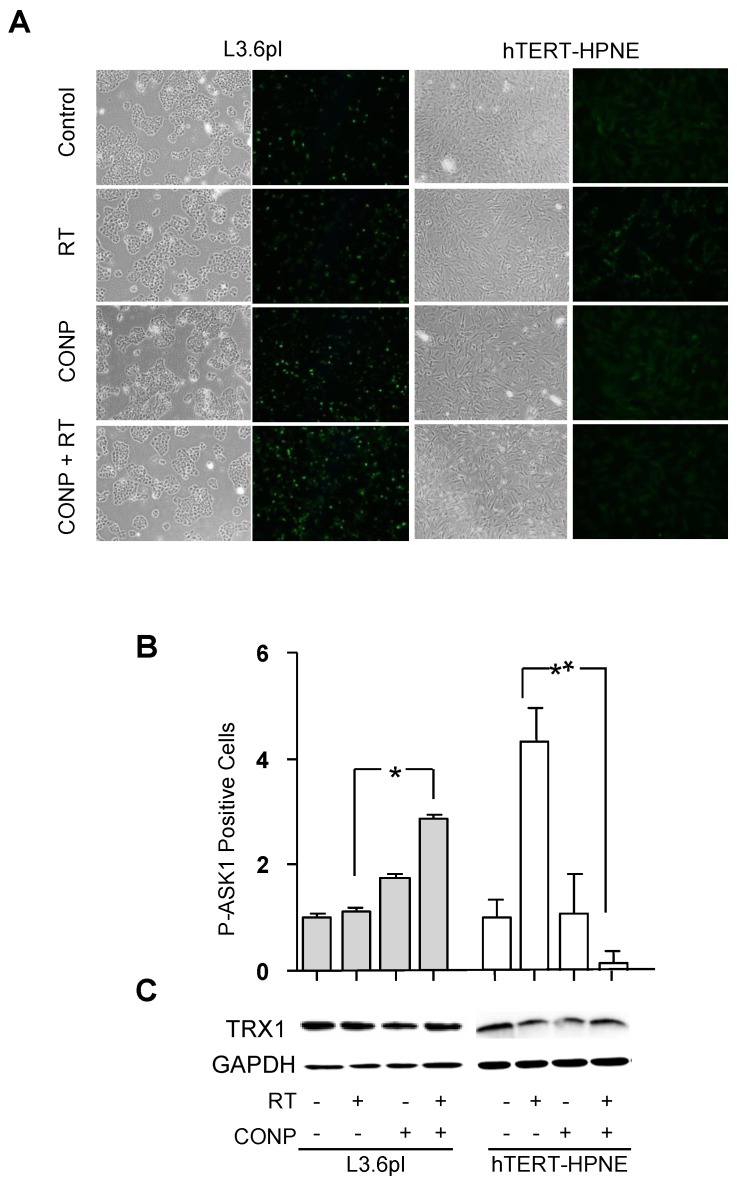
CONP Sensitization Increases RT-Induced ASK1 Activation Selectively in Pancreatic Cancer Cells. (**A**) The L3.6pl and hTERT-HPNE cells were pre-treated with 0 μM or 10 μM CONPs for 24 h followed by exposure to 0 Gy or 5 Gy RT. After 4 h, the cells were fixed, stained for p-ASK1 and analyzed. Representative phase contrast and fluorescent microscopic images are shown. (**B**) The number of fluorescent cells in 10 fields of view per condition was counted and normalized to untreated control group. (**C**) Unaltered TRX1 levels in an aliquot of the cells were determined by western blot. * *p* < 0.001; ** *p* < 0.0001.

The results described above also indicate a pro-apoptotic role of TRX1-JNK pathway in the CONPs and TR treated cancer cells. Apoptosis signaling kinase 1 (ASK1), a protein responsible for activating apoptotic signaling through JNK, is directly downstream of TRX1. The reductive form of TRX1 binds and masks ASK1 in its inactive state. Upon TRX1 oxidation, ASK1 dissociates and auto-phosphorylates become activated. Therefore, ASK1 phosphorylation provides the link between ROS-induced TRX1 oxidation and the activation of JNK to induce apoptosis [19]. ASK1 phosphorylation was determined after RT exposure in the pancreatic cells (Figure 4A,B).

L3.6pl cancer cells pre-treated with CONPs showed greater than a 1.5-fold (*p* < 0.0001) increase in ASK1 phosphorylation over RT alone. RT alone was not able to induce significant ASK1 activation in either pancreatic cancer cell line. CONPs alone, however, did induce mild ASK1 activation in the same cell. Similar results were obtained from the Panc1 cell line (data not shown). Conversely, the normal hTERT-HPNE cells showed roughly a 4-fold decrease in ASK1 phosphorylation when pre-treated with CONPs compared to RT alone. CONPs alone did not significantly alter ASK1 activation in normal pancreatic epithelial cells compared to mock-treated control levels. Of note, none of the treatments had a significant impact on TRX1 expression (Figure 4C).

### 2.5. CONPs Increase JNK Activation and Apoptotic Signaling In Vivo

**Figure 5:**
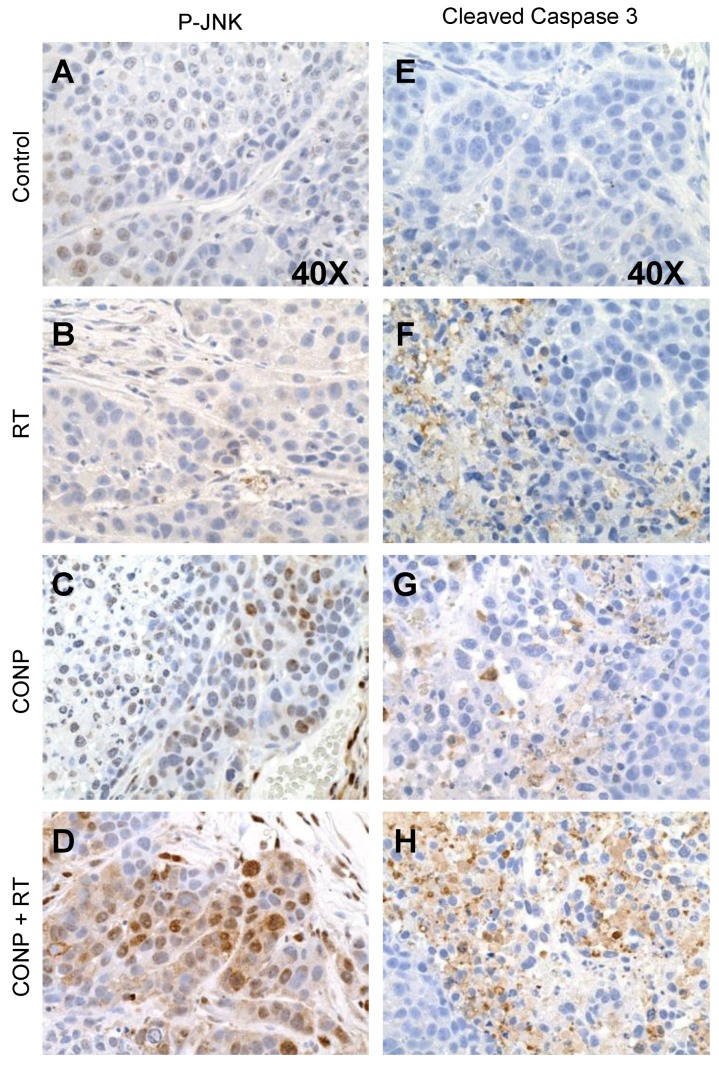
CONPs Enhance RT-Induced Activation of Apoptotic Signaling in the L3.6pl Tumor Cells in vivo. The pancreatic tumors were developed as described in the Materials and Methods section. Tumor tissues were collected at the time when mice were sacrificed and processed for IHC staining for P-JNK (**A**–**D**) and cleaved caspase 3 (**E**–**H**).

To corroborate the apoptotic signaling observed in vitro, we next tested the effect of combination therapy on JNK activation and apoptosis in pancreatic tumors established by implanted L3.6bl cells in nude mice. JNK activation (Figure 5A–D) and caspase 3 cleavage (Figure 5E–H) were determined in tumor sections from mice receiving only saline injections, RT alone, CONPs alone, or a combination of CONPs and RT. Results clearly show increases in activation of both JNK and caspase 3 (brown staining), in each of the treatment groups compared to saline control mice. However, it is also evident that the combination of CONPs and RT yielded the most significant increases in both JNK activation and caspase 3 activation (Figure 5D,H), compared with RT alone (Figure 5B,F) or CONPs alone (Figure 5C,G).

### 2.6. CONPs Are Unable to Induce Radiosensitization of Pancreatic Cancer Cells in the Absence of JNK Activity

**Figure 6:**
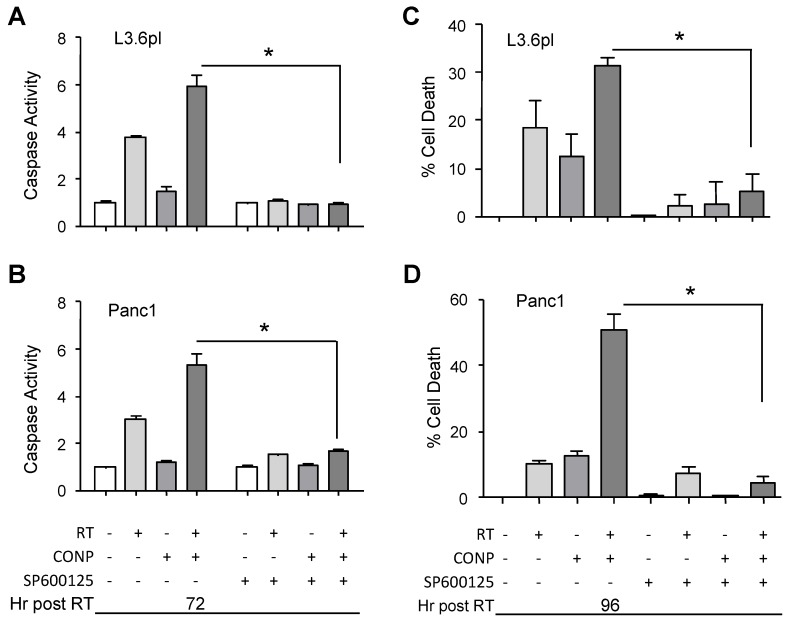
CONPs are Unable to Drive RT-Induced Apoptosis in the Absence of JNK Function. Cells were treated with or without 20 µM specific JNK inhibitor (SP600125) plus 0 or 10 µM CONPs for 24 h prior to exposure to 0 Gy or 5 Gy RT. Caspase 3/7 activation (**A**,**B**) and cell viability (**C**,**D**) were determined 72 and 96 h post RT, respectively. Results were normalized to untreated control group. The control viability value was set as 100% viability. The % cell death was calculated by subtracting % viability from 100%. * *p* < 0.001. (see Figure S2 for similar results obtained when JNK expression was knocked down).

To determine if JNK activation was the molecular driver of CONP-induced sensitization, we next used JNK inhibitors to block JNK activity in the cells exposed to RT, CONPs, or their combination. In the presence of a JNK inhibitor (SP600125), caspase 3/7 activation and sensitization to RT-induced cell death were completely mitigated in L3.6pl (Figure 6A,C) and Panc1 (Figure 6B,D) pancreatic cancer cells. Similar results were demonstrated with an independent JNK inhibitor (BI 78D3) (data not shown) as well as JNK silencing (Supplementary Figure S2), demonstrating that JNK function is essential for CONP-induced radiosensitization in pancreatic cancer cells.

### 2.7. CONPs Fail to Induced Radiosensitization of Pancreatic Cancer Cells in the Absence of TRX1 Oxidation

**Figure 7:**
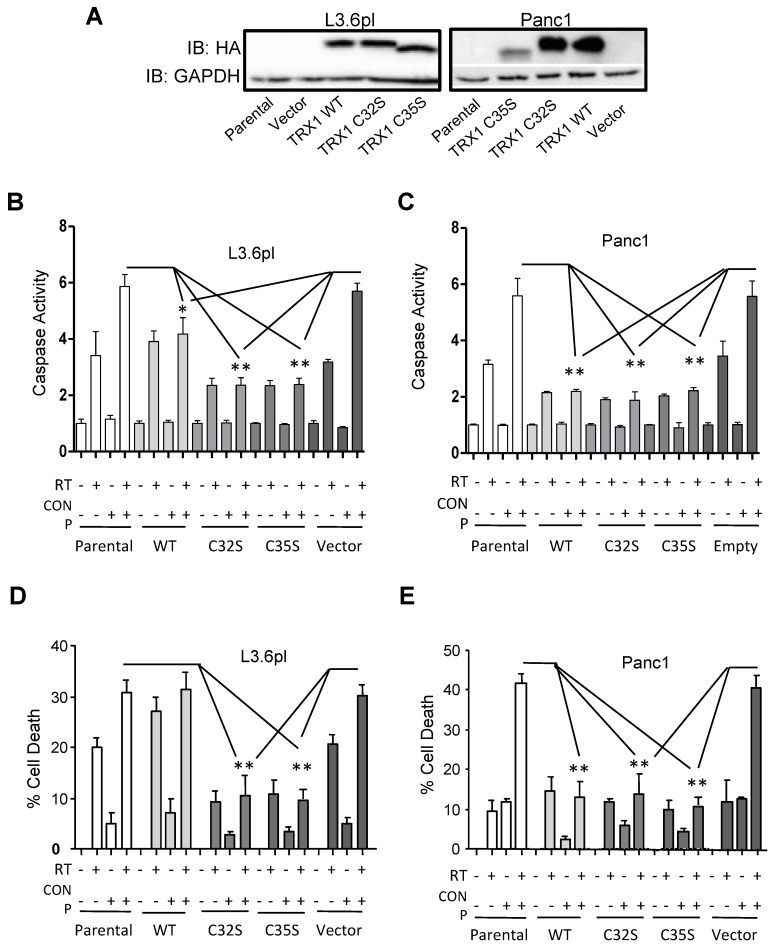
CONPs are Unable to Drive RT-Induced Apoptosis in the Absence of TRX1 Oxidation. The L3.6pl and Panc1 cells with stable expression of TRX1 (verified by western blot (**A**)) were treated with or without 10 µM CONPs for 24 h prior to 0 Gy or 5 Gy RT. Caspase 3/7 activation (**B**,**C**) and cell viability (**D**,**E**) were determined 72 and 96 h post RT, respectively. Results were presented as relative to untreated control group. The control viability value was set as 100% viability. The % cell death was calculated by subtracting % viability from 100%. * *p* < 0.001.

To confirm if the TRX1-ASK1 pathway is the critical activator of JNK and apoptosis in mediating radiosensitization by CONPs, we generated L3.6pl and Panc1 cell lines that stably expressed HA-tagged wild type TRX1, its C32S or C35S mutant, or the empty lentiviral vector. Stable TRX1 protein expression was confirmed by anti-HA western blot (Figure 7A). The C32 and C35 are required for TRX1 oxidation and subsequent release and activation of ASK1. Unlike the wild-type TRX1, the C32S and C35S mutants are unable to activate ASK1 and downstream JNK apoptotic signaling [20]. Indeed, these mutations significantly blocked the caspase activation (Figure 7B,C) and apoptotic induction (Figure 7D,E) by CONPs pre-treatment in both the L3.6pl and Panc1 cancer cells.

**Figure 8:**
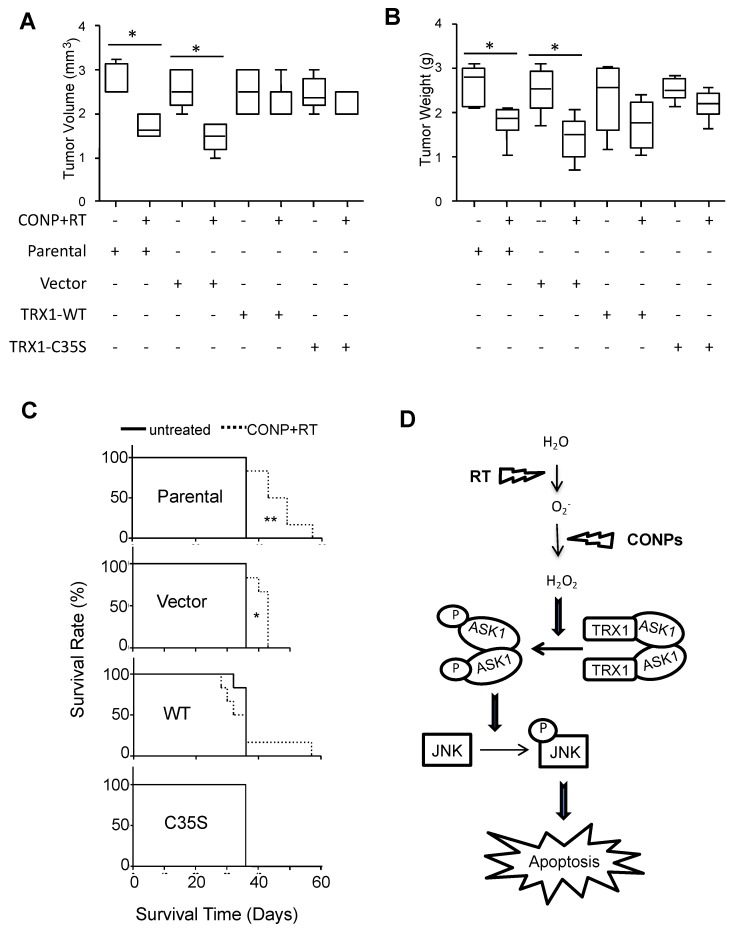
CONPs Fail to Sensitize Pancreatic Tumors to RT in the Absence of TRX1 Oxidation. The L3.6pl pancreatic cancer cell line overexpressing nothing (parental), empty vector, wild-type (WT) or mutant (C35S) TRX1 were implanted into nude mice followed by treatment with CONPs plus RT as described in the Materials and Methods section. At the time of death, the tumor volume and weight (**A**,**B**) as well as survival rate (**C**) were recorded. ** *p* < 0.01 with 10 days difference. * *p* < 0.01 with 7 days difference. (**D**) A model of mechanism of action.

The role of the TRX1-ASK1 pathway was further tested in vivo. As expected, the parental and vector control cells were highly responsive to the combination of CONPs and RT with much reduced tumor burden. When TRX1 was overexpressed, however, such responsiveness was lost (Figure 8A,B), consistent with that seen in vitro. Consequently, the combination treatment failed to extend the survival rate in the mice bearing the tumors with overexpressed TRX1 whereas lifespan of the mice bearing the tumors without overexpression was significantly extended compared the combination treatment to untreated control groups (Figure 8C).

## 3. Discussion

Our work identified the TRX1-ASK1-JNK apoptosis pathway as a novel CONPs responsive redox signaling that sensitize pancreatic cancer to RT (Figure 8D). In the absence of CONPs, the reduced form of TRX1 masks the ASKs from phosphorylation and activation. CONPs convert the radical superoxide to RT-produced hydrogen peroxide that oxidizes TRX1 and releases ASK1 for phosphorylation and activation. The subsequent activation of JNK by ASK1 triggers the apoptosis in the cancer cells.

This conclusion is consistent with our previous work showing that CONPs induce H_2_O_2_ production in pancreatic cancer cells [14]. It is further supported by our results demonstrating the requirement for the reduction of TRX1 and inactivation of ASK1 and JNK for the tumor insensitivity to RT, and this barrier is breached by CONPs that facilitate oxidation of TRX1 and subsequent activation of ASK1 and JNK for apoptosis (Figure 2, Figure 3, Figure 4 and Figure 5 and Figure S1). Indeed, blocking TRX1 oxidation or JNK activation prevents CONPs from sensitizing the cancer cells or tumors to RT (Figure 6, Figure 7 and Figure 8 and Figure S2).

The inherent overexpression of TRX1 by cancer cells (Figure 4C) indicates a higher than normal volume of total TRX1 pool. Hence, the pool of reduced TRX1 in the cancer cell available to inhibit ASK1 activation allows for the cell ability of resistance to RT. This could explain why forced overexpression of the wild-type TRX1 does not enhance the radiosensitization by CONPs (Figure 7). Nevertheless, the high level of TRX1 expression in the cancer cells does not seem to be a primary contribution to the CONPs-inducted radiosensitization since TRX1 expression in either pancreatic cancer cells or normal pancreatic epithelial cells does not change in response to treatment with RT, CONPs or their combination (Figure 4C). It is interesting that overexpression of the wild type TRX1 was more sensitive than the C32S and C35S mutants to the RT plus CONPs combination in vitro (Figure 7D) but the wild type and mutant TRX1 were equally resistant to the treatment in vivo (Figure 8A–C). This could be due to the difference in the cell growth environment between the in vitro tissue culture and the in vivo tumor microenvironment that differentially affects the oxidation of TRX1 mediated by CONPs. This notion can be tested in a 3-dimensional coculture of tumor cell with stromal cells mimicking tumor growth conditions in vivo with regulated oxygen supplies and pH value although a reliable device for measuring acidity inside tissues remains unavailable. ASK1-mediated sustained activation of JNK has been shown to be required for ROS-induced apoptosis, with ASK1 knockout cells being substantially more resistant to H_2_O_2_ [21]. These results suggest that the RT-CONPs combination therapy primarily targets TRX1 oxidation rather than its expression to enhance apoptosis.

In addition to JNK apoptotic pathway, there are a variety of other pro- and anti-apoptotic pathways such as the AKT, ERK, and p53 that play key roles in pancreatic cancer. AKT activation has been linked to cancer cell growth, survival, and proliferation [22], as well as correlated to the invasiveness of pancreatic cancers [23]. ERK1/2 has been shown to protect pancreatic cancer cells from apoptosis and promote progression through the cell cycle [24]. Both AKT and ERK1/2 signaling frequently become over-activated in pancreatic cancers. The P53 tumor suppressor normally plays a key role in inducing cell death in response to significant DNA damage, but becomes inactive in more than 60% of pancreatic cancers [25]. While AKT [26], ERK1/2 [27], and p53 [28] may all become active in response to ROS, our results showed that none of them, unlike JNK, was involved in the cellular response to CONP-driven RT-induced ROS (Figure 1). Although JNK activation can be both pro- and anti-apoptotic, depending upon cellular context [29], it is generally accepted that it induces apoptotic effects in cancer cells exposed to RT [30]. However, while JNK activation appears to be the primary redox-responsive effect in pancreatic cancer, it is possible that apoptotic pathways other than the JNK pathway could play a main role in other types of cancer in response to RT-CONPs combination therapy.

CONPs have been shown to be toxic to various types of cancer cells [14,15,31] by inducing stress oxidation, glutathione oxidation, lipid peroxidation, and membrane damage [14,31]. However, whether CONPs plays a role in DNA damage and whether such a role contributes to radiosensitization of cancer cells have never been reported. Our results clearly demonstrate that CONPs and RT combination does not have a synergistic effect on the cancer cells (Figure 1), excluding mitotic catastrophe as a main radiosensitization mechanism.

Despite the incapability of sensitizing to RT-induced DNA damage-mitotic catastrophe in cancer cells, CONPs alone induce a low level of DNA damage in the cancer cells but not the normal cells. Further, CONP treatment dramatically decreased the amount of initial DNA damage in normal pancreatic epithelial cells exposed to RT (Figure 1). This difference may be attributable to the differential activity of CONPs at differing pH that determines whether CONPs perform like SOD to attack or catalase to protect the cell [14]. Therefore, only under acidic conditions found in cancer cells, CONPs may be able to directly induce a low level of DNA damage. Combining with the effect of CONPs on activation of the TRX1-ASK1-JNK apoptotic pathway predominantly in the cancer cells, our results further support the potential anticancer therapeutic applications of CONPs as a novel radiation adjuvant.

## 4. Materials and Methods

### 4.1. Cell Culture

Normal (non-tumorigenic) pancreatic cells (hTERT-HPNE) were obtained from American Type Culture Collection (ATCC, Manassas, VA, USA) and maintained in 3:1 glucose free DMEM:M3 Base medium, supplemented with 10% fetal bovine serum, 100 µg/mL gentamycin, and 1 g/L dextrose. The human pancreatic cancer cell lines Panc1 (ATCC) and L3.6pl were cultured in DMEM. Both cell mediums were supplemented with 10% fetal bovine serum and 100 µg/mL penicillin-streptomycin mixture (GIBCO, Gaithersburg, MD, USA), with cells maintained at 37 °C and 5% CO_2_.

### 4.2. Generation of Stable Cell Lines

Wild type TRX1 and its C32S and C35S mutants (Addgene 20183, 21284 and 21285) [20] were transferred into the pKH3 vector to add an HA-Tag [32,33] and then into the lentiviral pLVPZ vector encoding puromycin resistance. As previously described [34], the lentivirus was produced by transfecting 293FT cells with the vectors, and the media collected from the transfected 293FT cells were used to infect L3.6pl and Panc1 cells for three repeated times. Positively infected cells were then selected with and maintained in medium supplemented with 1 µg/mL puromycin. Stable TRX1 protein expression was confirmed by anti-HA western blot.

### 4.3. Comet Assay

L3.6pl, Panc1 or hTERT-HPNE cells were plated in 10 cm dishes and left to attach overnight. Cells were then treated with 0 or 10 µM CONPs. 24 h later, cells were exposed to 0 or 5 Gy RT using a 160-kV cell culture and small animal irradiator (Kimtron Inc., Woodbury, CT, USA). Cells were trypsinized immediately following RT and subjected to single cell electrophoresis (CometAssay^®^, Trevigen, Gaithersburg, MD, USA) as described previously [35] according to the manufacturer’s protocol, including control cells with predetermined amounts of DNA damage purchased from Trevigen with the kit.

### 4.4. Phospho-ELISA

L3.6pl cells were seeded into 10 cm dishes and left to attach overnight. Cells were then treated with 0 or 10 µM CONPs followed by exposure to 0 or 5 Gy RT 24 h later. 72 h post RT, cells were collected and cell lysate was subjected to screening with the PhosphoELISArray Multi-Analyte Kit for key pathways associated with human cancer according to the manufacturer’s protocol (SA Biosciences, FEM-5001, Frederick, MD, USA).

### 4.5. Antibodies, Drugs, and Western Blot

Primary antibodies (TRX #2429, p-ASK1 #3765, p-JNK1/2 #9251, JNK1/2 #9252, Cleaved Caspase-3 #9664, HA-Tag #3724, GAPDH #5174) were purchased from Cell Signaling Technology (Danvers, MA, USA). Secondary antibodies (Alexa Fluor^®^ 488 anti-rabbit #A-11034 and anti-rabbit #sc-2004) were purchased from Invitrogen (Carlsbad, CA, USA) and Santa Cruz Biotechnology (Santa Cruz, CA, USA), respectively. CONPs were purchased from the NanoScale Corporation (Manhattan, KS, USA) or synthesized as previously described [14,36]. For western blotting, cells were plated in 10 cm dishes and left to attach overnight. Media was then removed and replaced with media containing 0 or 10 µM CONPs. 24 h later, cells were exposed to 0 or 5 Gy RT with cell lysates collected 24–72 h after radiation. Proteins were separated on 12.5% SDS-PAGE gels, transferred to nitrocellulose membranes using the iBlot system (Invitrogen, Carlsbad, CA, USA), and blotted following standard procedures. The chemiluminescence in the blots were recorded and quantified by selecting the same exposure duration for all the membranes using Image Lab 3.0 (Bio-Rad, Hercules, CA, USA) as described previously [35].

### 4.6. P-ASK1 Immunofluorescent Staining

hTERT-HPNE, Panc1, and L3.6pl cells were seeded onto glass coverslips and let attach overnight. Media was then removed and fresh media containing 0 or 10 µM CONPs was added. 24 h later, cells were exposed to 0 or 5 Gy RT. 4 h post RT exposure, coverslips were washed 2 × in ice-cold phosphate-buffered saline (PBS) (5 min) and fixed in 10% paraformaldehyde (20 min) at room temperature. Coverslips were then washed 3 × in ice-cold PBS (5 min) and permeabilized with ice-cold acetone (5 min) at −20°C. Subsequent to 3 more washes (5 min) in ice-cold PBS, coverslips were blocked (5% bovine serum albumin (BSA) in PBS) for 1 h at room temperature followed by incubation in primary p-ASK1 antibody (1:50–1:100) at 4 °C overnight (Cell Signaling, #3765). Coverslips were then washed 3 × (5 min) in ice-cold phosphate-buffered saline with Triton (PBST), followed by incubation Alexa Fluor^®^ 488 anti-rabbit secondary (1:100–1:200) at room temperature for 1 h (Invitrogen). Finally, coverslips were washed 3 × (5 min) in ice-cold PBS and mounted using ProLong^®^ Gold antifade reagent with DAPI. A 10 × objective on a Nikon E400 microscope (Nikon Instruments, Melville, NY, USA) was used to capture random fields on each slide (slides stained in triplicate). P-ASK1 positive cells were quantified using Image J software (NIH, Bethesda, MD, USA).

### 4.7. Analysis of Caspase Activation and Cell Viability

hTERT-HPNE, Panc1, and L3.6pl cells were seeded onto white-walled, clear-bottomed, 96 well plates (2,000 cells/well) and left to attach overnight. Media was then removed and media containing 0 or 10 µM CONPs was added. 24 h later, cells were exposed to 0 or 5 Gy radiation. At 24–48 h post radiation, caspase 3/7 activation was determined via Caspase-Glo^®^ 3/7 Assay (Promega, Madison, WI, USA) following the manufacturer’s protocol. Caspase activity was normalized to cell viability, which was determined with CellTiter-Glo^®^; Luminescent Cell Viability Assay (Promega). Data was normalized to control (untreated) caspase levels for graphing. Cell viability was determined using WST-1 assay as previously reported [35,37].

### 4.8. JNK Inhibitor Studies

Panc1 and L3.6pl cells were seeded into 10-cm dishes and left to attach overnight. Media was then removed and replaced with media containing 0 or 10 µM CONPs and 20 µM specific JNK inhibitor (EMD #420119) [38], added at the same time. Radiation treatment (0 or 5 Gy) and the subsequent analysis of caspase activation (Caspase-Glo^®^ 3/7 Assay, Promega) normalized to viability (CellTiter-Glo^®^ Luminescent Cell Viability Assay, Promega) were completed by the same protocol as previously described. Additionally, the impact of the JNK inhibitor to mitigate radiation-induced cell death and CONP-driven radiosensitization was also determined from the CellTiter-Glo Luminescent Cell Viability Assay (Promega) data, which was normalized to control levels.

### 4.9. Orthotopic Xenograft of Human Pancreatic Cancer Cells in Athymic Mice

Experiments were performed essentially as described in our previous report [14]. Briefly, female athymic mice of 4 to 6 weeks of age (NCI-nu) were transported to our vivarium, housed and maintained in specific pathogen-free rooms. The project was approved by the institutional animal care and use committee (IACUC protocol #11–22) in accordance with the American Association for Accreditation of Laboratory Animal Care and current regulations and standards of the United States Department of Agriculture, Department of Health and Human Services, and the National Institute of Health. The mice were used in accordance under institutional guidelines with approved IACUC protocol. To develop tumors, L3.6pl cells, as well as L3.6pl cells stably expressing wild type TRX1, C32S mutant TRX1, and the empty pLVPZ vector, were harvested from culture dishes and injected as previously described [39,40,41].

### 4.10. Therapeutic Approach for Human Pancreatic Tumors Bearing Athymic Mice

The L3.6pl human pancreatic cancer cells with or without ectopic overexpression of wild type TRX1, C35S TRX1, and the empty pLVPZ vector were implanted into athymic mice. The mice were then grouped by random and treated with CONPs, RP, CONPs in combination with RP or saline. Tumor monitoring and subsequent analyses by liquid displacement and immunohistochemical staining were done as we previously described [14].

### 4.11. Statistical Analysis

Data are presented as mean ± standard deviation with a minimum of three observations per group. Unpaired, paired or single sample Student’s *t*-test was applied using the GraphPad Prism software (GraphPad Software, Inc., La Jolla, CA, USA) with the Bonferroni correction for the multiple comparisons. Animal survival was analyzed using Kaplan-Meier survival plot. Significance was determined by the alpha level of 0.05.

## 5. Conclusions

In summary, this work identified the TRX1-ASK1-JNK redox-sensing pathway to be a novel mechanism used by CONPs to re-activate the apoptotic mechanism in the human pancreatic cancer cells in response to RT. These findings provide further insight into the cellular implications of CONP-based therapies, especially in conjunction with RT or other potential radical inducing agents. Overall, this work supports the pursuit of CONPs as a novel tumor tissue sensitizer to increase the therapeutic index of RT to benefit pancreatic and potentially other types of cancer patients.

## Supplementary Materials

The following are available online at http://www.mdpi.com/2072-6694/10/9/303/s1, Supplementary Figure S1, CONP Pre-Treatment Drives RT-Induced JNK2 Activation in Pancreatic Cancer Cells (Supplemental to Figure 2B). Supplementary Figure S2, CONPs Do Not Drive RT-Induced Apoptosis in the Absence of JNK Expression (Supplemental to Figure 6).
