# Supplementary material for: Cerium Oxide Nanoparticles Sensitize Pancreatic Cancer to Radiation Therapy through Oxidative Activation of the JNK Apoptotic Pathway
Source: Cancers (Basel). 2018 Sep 1;10(9):303. doi: 10.3390/cancers10090303 (PMC6162528; doi:10.3390/cancers10090303)
Supplement: Supplementary file 1 [file cancers-10-00303-s001.pdf]

Cerium Oxide Nanoparticles Sensitize Pancreatic Cancer to Radiation Therapy through Oxidative Activation of the JNK Apoptotic Pathway

Melissa S. Wason, Heng Lu, Lin Yu, Satadru K. Lahiri, Debarati Mukherjee, Chao Shen, Soumen Das, Sudipta Seal and Jihe Zhao

Supplementary Materials

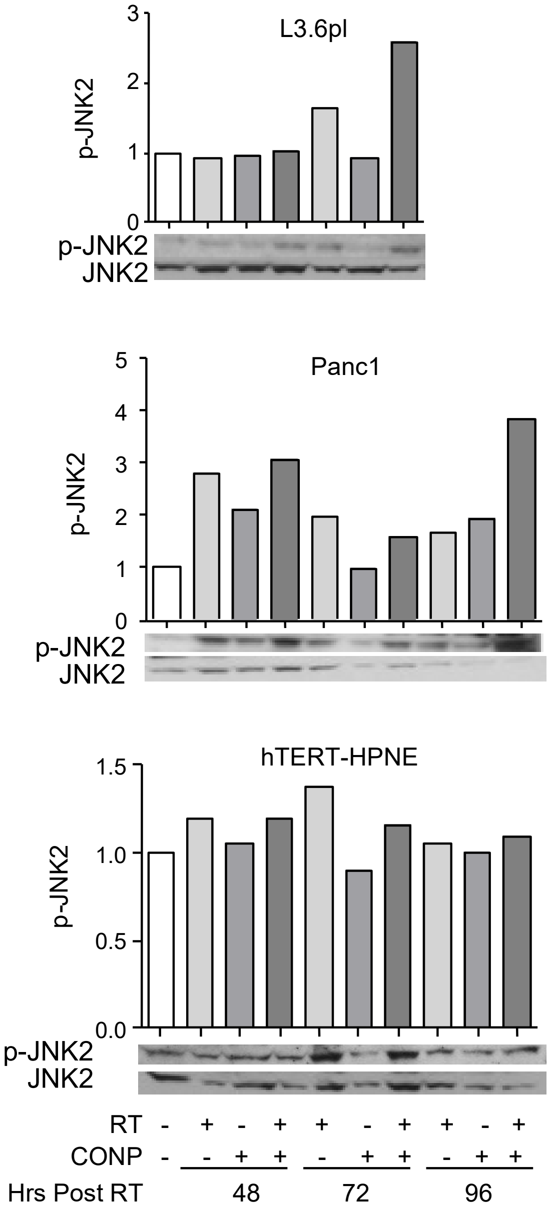

Figure S1. CONP Pre-Treatment Drives RT-Induced JNK2 Activation in Pancreatic Cancer Cells. Cells were treated with 10 μM CONPs for 24 h followed by exposure to 0 or 5 Gy RT for additional hours as indicated prior to western blot. (Supplemental to Figure 2B.)

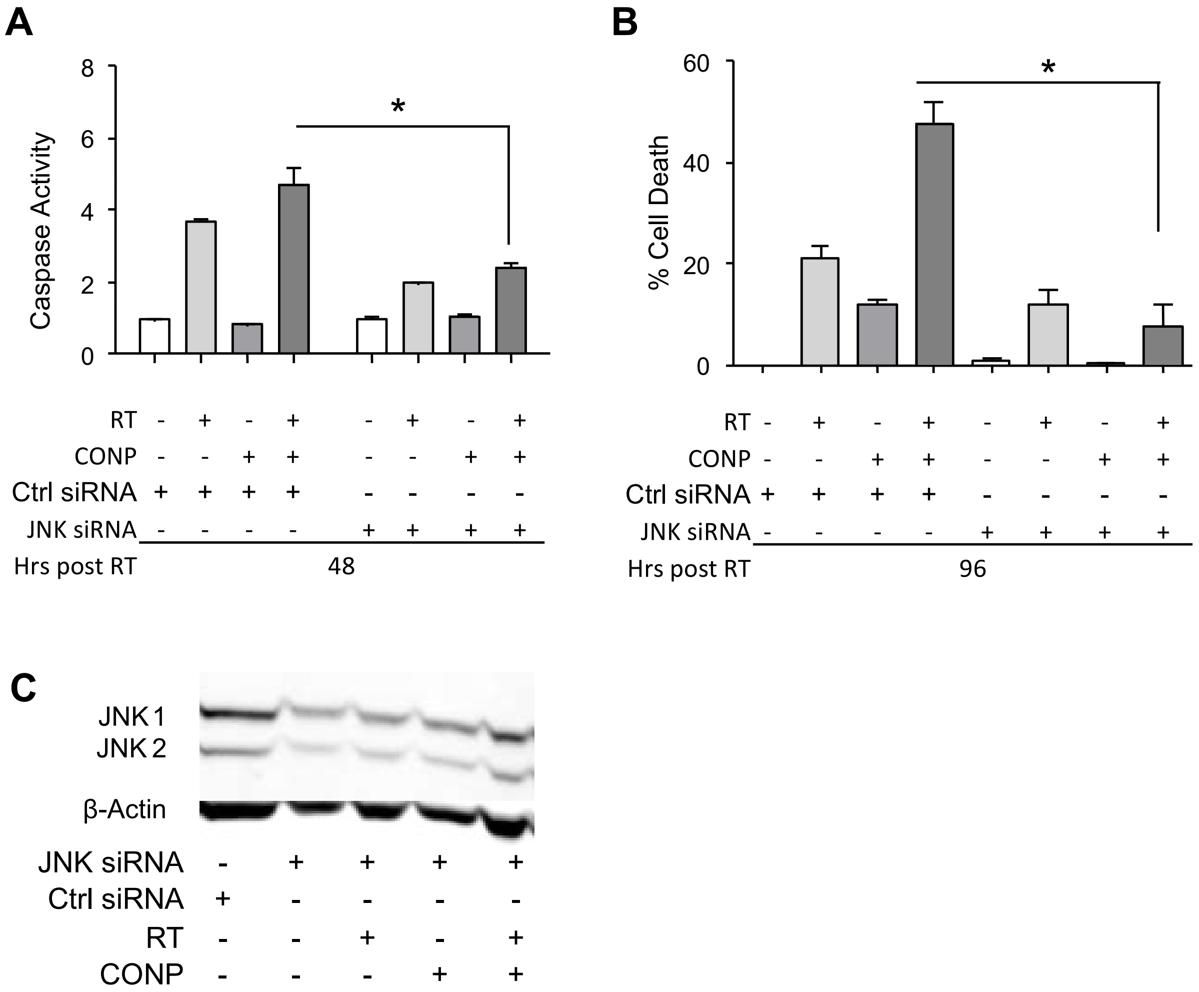

Figure S2. CONPs Do Not Drive RT-Induced Apoptosis in the Absence of JNK Expression. Panc1 cells were transfected with siRNA for 24 hours and treated with 0 or 10 μM CONPs for additional 24 hours followed by exposure to 0 or 5 Gy RT. After the 24 and 96 hours, the cellular caspase activity (**A**) and viability (**B**) were determined, respectively. Results were normalized to untreated control siRNA group. The control viability value was set as 100% viability. The % cell death was calculated by subtracting % viability from 100%. Effective JNK silencing (**C**) was confirmed by western blot for total JNK protein levels 24 hours post siRNA treatment. (Supplemental to Figure 6.)
